# Use of Plerixafor to Mobilize a Healthy Donor Infected with Influenza A

**DOI:** 10.4274/tjh.2017.0304

**Published:** 2018-05-25

**Authors:** Mahmut Yeral, Pelin Aytan, Can Boğa

**Affiliations:** 1 Başkent University Adana Practice and Research Center, Adult Bone Marrow Transplantation Center, Adana, Turkey

**Keywords:** Plerixafor, Influenza A, Healthy donor

**To the Editor,**

The combined use of plerixafor and granulocyte-colony stimulating factor (G-CSF) improves mobilization in poor mobilizers. However, there are limited data available on the use of plerixafor in healthy donors [1,2]. The effects of influenza A infection on stem cell mobilization are not known. 

A 46-year-old male was selected as an HLA-matched donor for a patient diagnosed with acute myeloid leukemia (AML). Donor assessment was performed in accordance with the standard operating procedure prepared for JACIE (SOP: BMT-CU-006, Donor Assessment and Safety). The donor was given 10 mg/kg/day G-CSF. He developed a dry persistent cough, chills, fever of 39 °C, fatigue, and flu-like symptoms on day 3 of G-CSF administration. The donor was considered to have an upper respiratory tract infection, which could not be attributed to only G-CSF administration. The family members of the donor were found to have similar symptoms. Thus, blood and urine cultures were obtained and he was started on levofloxacin in addition to paracetamol; G-CSF was continued. A respiratory tract virus panel was performed on a nasal smear using a PCR-based technique. The peripheral blood leukocyte count was 22,000/µL but CD34+ cells represented just 0.07% of all cells (11/µL) on day 5 of G-CSF administration; this was considered to reflect “poor mobilization”. Therefore, 0.24 mg/kg plerixafor was administered “just in time,” in addition to G-CSF, on night 5, after the donor had been given all necessary information and informed consent had been obtained. Two hours after the 11^th ^dose of G-CSF, the leukocyte count was 45,000/µL, of which 0.33% (148/µL) were CD34+ cells. Peripheral stem cell apheresis was performed using the Donor Spectra Optia Apheresis System (Terumo BCT, Lakewood, CO, USA). A total of 15.20x10^8^ nuclear cells/kg were collected. The product contained 3.92x10^6^ CD34+ cells/kg, 14.91x10^7^ CD3+ cells/kg, 17.36x10^7^ CD19+ cells/kg, and 7.17x10^7^ CD56+ cells/kg. G-CSF was discontinued after an adequate number of stem cells had been collected, but the fever persisted. Oseltamivir at 150 mg twice daily was then prescribed for the donor because the respiratory tract virus panel examination revealed influenza A infection. The fever became controlled 24 h after oseltamivir administration. The plerixafor procedure was considered to have permitted “sufficient mobilization” in a healthy donor who could not be mobilized with G-CSF probably because of his influenza infection.

Many factors including age, sex, body mass index, baseline leukocyte count, and comorbid conditions may compromise mobilization [3]. Although certain viral infections may cause poor mobilization, data on the influence of influenza in this context are rather limited [4]. Cytokine production or cytokine storm developing during influenza infection may be presumed to impair stem cell mobilization [5]. A combination of G-CSF and plerixafor can be used to treat mobilization failure and is usually well tolerated [6,7]. The only option upon stem cell mobilization failure with G-CSF is bone marrow harvesting. Our donor was given plerixafor “just in time”; he had an active infection and did not consent to bone marrow harvesting. While plerixafor is usually used for mobilization in lymphoma or myeloma patients, literature data are available about its use in allogeneic settings [8]. Stem cells in numbers adequate for safe transplantation were collected in a single procedure. 

This report indicates that influenza A may suppress the hematopoietic system, negatively affecting stem cell mobilization. The problem may be overcome by plerixafor administration.
